# Iron-Dependent Autophagic Cell Death Induced by Radiation in MDA-MB-231 Breast Cancer Cells

**DOI:** 10.3389/fcell.2021.723801

**Published:** 2021-10-14

**Authors:** Shumei Ma, Xinxin Fu, Lin Liu, Yi Liu, Hao Feng, Heya Jiang, Xiaomei Liu, Rui Liu, Zhenzhen Liang, Mengke Li, Zhujun Tian, Boqi Hu, Yongheng Bai, Bing Liang, Xiaodong Liu

**Affiliations:** ^1^School of Public Health and Management, Wenzhou Medical University, Wenzhou, China; ^2^NHC Key Laboratory of Radiobiology, Jilin University, Changchun, China; ^3^China-Japan Union Hospital of Jilin University, Changchun, China; ^4^Key Laboratory of Diagnosis and Treatment of Severe Hepato-Pancreatic Diseases of Zhejiang Province, The First Affiliated Hospital of Wenzhou Medical University, Wenzhou, China; ^5^School of Nursing, Jilin University, Changchun, China

**Keywords:** lysosome membrane permeabilization (LMP), radiation, breast cancer, reactive oxygen species (ROS), autophagy, iron

## Abstract

In radiation oncology, ionizing radiation is used to kill cancer cells, in other words, the induction of different types of cell death. To investigate this cellular death and the associated iron accumulation, the transfer, release, and participation of iron after radiation treatment was analyzed. We found that radiation-induced cell death varied in different breast cancer cells and autophagy was induced in MDA-MB-231 and BT549 cells (triple negative breast cancer cell line) rather than in MCF-7 and zr-75 cells. Iron chelator deferoxamine (DFO), the autophagy inhibitor 3MA, silencing of the autophagy-related genes ATG5, and Beclin 1 could decrease radiation induced cell death in MDA-MB-231 cells, while inhibitors of apoptosis such as Z-VAD-FMK, ferroptosis inhibitor ferrostatin-1 (Fer-1), and necroptosis inhibitor Necrostatin-1 showed no change. This suggests the occurrence of autophagic cell death. Furthermore, we found that iron accumulation and iron regulatory proteins, including transferrin (Tf), transferrin receptor (CD71), and Ferritin (FTH), increased after radiation treatment, and the silencing of transferrin decreased radiation-induced cell death. In addition, radiation increased lysosomal membrane permeabilization (LMP) and the release of lysosomal iron and cathepsins, while cathepsins silencing failed to change cell viability. Radiation-induced iron accumulation increased Reactive oxygen species (ROS) generation via the Fenton reaction and increased autophagy in a time-dependent manner. DFO, *N*-acetylcysteine (NAC), and overexpression of superoxide dismutase 2 (SOD2) decreased ROS generation, autophagy, and cell death. To summarize, for the first time, we found that radiation-induced autophagic cell death was iron-dependent in breast cancer MDA-MB-231 cells. These results provide new insights into the cell death process of cancers and might conduce to the development and application of novel therapeutic strategies for patients with apoptosis-resistant breast cancer.

## Introduction

Breast cancer is the most common tumor in women, and the metastasis further increases the malignancy with extremely high mortality. Clinically, specific subtypes of breast cancer are defined by their histopathological appearance and expression of hormone receptors and growth factors [namely, the estrogen receptor (ER), the progesterone receptor (PR) and human epidermal growth factor receptor 2 (HER2; also known as ERBB2]. Both genetic and non-genetic risk factors influence breast cancer development. At present, radiotherapy remains an important cornerstone of breast cancer therapy.

Radiation induces molecular damage directly or indirectly, either the radiation damages the DNA molecule directly and disrupts the molecular structure, or the radiation ionizes the water molecules and creates free radicals such as hydroxyl (HO•) and alkoxy (RO_2_•) radicals. Free radicals then lead to macromolecular damages (Kunwar and Priyadarsini, 2011; Desouky et al., 2015; Olcina and Giaccia, 2016).

The Nomenclature Committee on Cell Death (NCCD) states that cell death consists of programmed and non-programmed cell death, or typical and atypical cell death with different features and mechanisms (Kroemer et al., 2009). The pathways of cell death are diverse in breast cancer, but it is mainly through the apoptosis pathway to kill tumor cells in clinical treatment. The phenomenon of multidrug resistance (MDR) caused by tumor cell apoptosis tolerance becomes a difficult problem in the treatment of breast cancer. Therefore, it is essential to find new non-apoptotic pathways for treatment. Our previous data showed that in breast cancer cells, iron could participate in the regulation of ferroptosis and autophagic cell death after treatment with siramesine and lapatinib in the early and later stages, respectively (Ma et al., 2016). Considering the important roles of Reactive oxygen species (ROS) in radiation-induced molecular damage and cell death, and that high intracellular levels of unbound iron can contribute to the redistribution of different ROS via the Fenton’s reaction (Dixon and Stockwell, 2014), we designed this study to determine iron-mediated cell death after radiation from different aspects, including iron accumulation and release from lysosomes, the import and export of iron, iron-mediated ROS production, and the feedback between Lysosomal Membrane Permeability (LMP) and iron accumulation.

Lysosomal membrane permeabilization is a prominent feature of lysosomal dysfunction (Rodríguez-Muela et al., 2015; Wang et al., 2018). LMP is induced by magnanimous of distinct stimuli factors including ROS, lysosomotropic compounds with detergent activity as well as some endogenous cell death effectors such as Bcl2-Associated X protein (Bax). Ionizing radiation can induce LMP by directly breaking the lysosomal membrane and consequently, lysosomal iron and cathepsins release into the cytoplasm (Persson et al., 2005; Kurz et al., 2008).

Lysosomes are involved in the autophagic turnover of organelles and long-lived proteins, including iron-rich macromolecules, which makes lysosomes an iron pool. When iron is released into the cytoplasm, it will be associated to ferritin for further use in the synthesis of iron-containing biomolecules (Muñoz et al., 2011). Thus, autophagy is the key to balance the redox status via its ability to increase lysosomal iron levels as well as the autophagocytosis of iron-binding proteins.

Iron regulatory proteins participate in iron transport (Kühn, 2015). Ferroportin (FPN), located on the plasma membrane, conducts as the major iron efflux transporter to release Fe^2+^ from cells and a critical control site for recycling iron according to its needs; its expression on the plasma membrane is controlled mainly by the small peptide hepcidin (Linder, 2013). Transferrin and transferrin receptors are another pair of iron-regulatory proteins. The transferrin receptor mediates most of the cellular iron uptake by binding iron-transferrin to the cell surface, which is then internalized by receptor-mediated endocytosis (MacKenzie et al., 2008).

Iron catalyzes and participates in Fenton’s reaction, yielding extremely reactive hydroxyl radicals and enhancing ROS generation (Persson et al., 2005; Dixon and Stockwell, 2014). Iron accumulation probably has great significance to the overall sensitivity of cells to resistance to oxidative damage and, as argued herein, ionizing radiation (IR). In this study, the roles of Iron-Dependent autophagic cell death induced by radiation were investigated.

## Materials and Methods

### Reagents and Antibodies

Trypan blue solution (Prod. No. T8154), Prussian blue soluble (Prod No.03899), FeCl_3_⋅6H_2_O (Prod No.157740), Deferoxamine (Prod No. D9533), Ferrostatin-1 (Prod. No. SML0583), ammonium chloride (NH_4_Cl) (Prod. No. 254134), *N*-Acetyl-L-cysteine (Prod No. N7250), and phosphatase inhibitor cocktails 2 &3 (Prod. No. P5726 & P0044) were from Sigma-Aldrich (St. Louis, MO, United States). A protease inhibitor cocktail (ref no. 11836 153 001) was from Roche Diagnostics (Basel, Switzerland). The siRNAs against Atg5 (sc-41445), Becn1 (sc-29797), transferrin (sc-37176), ferroportin (sc-60663), CD71 (sc-37070), Cathepsin L (sc-29938) and Control siRNA-A (Sc-37007) were purchased from Santa Cruz Biotechnology (Dallas, TX, United States). Primary antibodies: anti-Atg5 (#2630), anti-Beclin-1 (#3738), anti-FTH1(#4393) anti-CD71 (#13113), were purchased from Cell Signaling Technology (Danvers, MA United States), anti-Transferrin (ab9538), anti-Cathepsin L (ab103574), anti-LAMP2 (ab25447) and anti-SLC40A1 (ab85370) were from AbCam (Cambridge, United Kingdom), and anti-actin from Sigma-Aldrich (Prod. No. A3853). Secondary antibodies: goat anti-rabbit IgG (H + L)-HRP conjugate (Cat. No. 170-6515) and goat anti-mouse IgG (H + L)-HRP conjugate (Cat. No. 170-6516) were obtained from Bio-Rad Laboratories (Mississauga, ON, Canada). Phen Green SK, diacetate (Cat. No. P14313), LysoTracker (cat: L-7526) and Dihydroethidium (Cat: D-1168) from Life Technologies (Thermo Fisher Scientific, Waltham, MA, United States). Opti-MEM I reduced serum medium (cat: 31985-070) from GIBCO-Life Technologies (Thermo Fisher Scientific).

### Cell Culture

The breast cancer cell lines MCF-10A, MDA-MB-231, zr-75 and MCF-7 were obtained from Beijing cell bank of Chinese Academy of Sciences and periodically tested for the absence of mycoplasma. The cell lines were grown in a suitable condition according American type culture collection (ATCC) guideline, supplemented with 100 units of penicillin per ml plus 100 μg of streptomycin per ml (cat 10378016, Life Technologies) and 10% fetal bovine serum, in a humidified 5% CO_2_, 37°C incubator. Cells were treated for various times in the absence and presence of a chemical inhibitor.

### Radiation

An X-ray generator (X-RAD 320 ix, Precision X-ray Inc., North Branford, CT, United States) was utilized to deliver radiation at a dose rate of 1.0 Gy/min.

### Colony Formation Assay

Cells were trypsinized to generate single cell suspension and seeded in 6-well plates at 500 cells per well. 24 h later, the cells were irradiated with different dose, as 0, 2, 4, 6, 8 Gy. After incubation for 14 days, colonies were fixed in anhydrous ethanol, stained with crystal violet and the number of colonies containing at least 50 cells was counted. The colony survival fraction was calculated for each treatment.

### Acridine Orange Staining for Lysosomal Membrane Permeability

Cells were stained with acridine orange (AO) according to the manufacturer’s instructions (AO; Life Technologies, Carlsbad, CA, United States) for 10 min, then washed twice in Dulbecco’s phosphate-buffered saline (DPBS). Lysosomes were visualized by monitoring red signals using an excitation filter of 460 nm (450–480 nm) and a long-pass > 515 nm emission/barrier filter. The cytoplasm showed green fluorescence, and the lysosome showed red fluorescence. AO leaks quickly from late endosomes and lysosomes and partially shifts the fluorescence from an orange fluorescence to a diffuse, green cytoplasmic fluorescence.

### Dihydroethidium Staining for the Detection of Reactive Oxygen Species

Reactive oxygen species generation was determined by flow cytometry with Dihydroethidium staining (DHE, D-1168). DHE could be oxidized by ROS into 2-hydroxyethidium (2-HE) (emission at 605 nm) and fluoresces red. The samples were collected and stained with 5 μM DHE and then incubated in the dark, inside the water bath at 37°C for 15 min. The cell suspension was then transferred to a 5 ml FACS tube and analyzed on a flow cytometer within 10 minutes using Cell Quest software (BD Biosciences).

### Determination of Mitochondrial Membrane Potential and Reactive Oxygen Species

After treatment, cells were trypsinized and then harvested, washed, and resuspended together with their supernatant in PBS. 3,3-Dihexyloxacarbocyanineiodide [DiOC6(3)] was added at 40 nM final concentration for ΔΨm and MitoSOX at 1 μM for superoxide anion. Most of the time double staining was done in order to assay simultaneously cell viability, with propidium iodide (PI, stock solution, 1 mg. mL-1) for DiOC6(3) and with TO-PRO-3 iodide (stock solution, 1 mg. mL-1) for MitoSOX. A supplemental double staining was used for the distinction between viable, apoptotic, and necrotic cells with YO-PRO-1/PI (Vayssiere et al., 1994; Zamzami et al., 1995).

### Prussian Blue Staining for Iron Accumulation

Prussian blue staining was used to detect the presence of iron oxide particles. Cells were fixed in 4% paraformaldehyde for 30 min, after three washes with PBS, incubated for 30 min with Prussian blue (10 mg/ml). Labeled cells were examined under a light microscope to determine intracellular iron oxide distribution.

### Iron Assay

Intracellular chelatable iron was determined using the fluorescent indicator phen green SK, the fluorescence of which is quenched by free iron particles. Samples were collected and stained with 0.2 μM phen green SK and then were incubated in the dark in a water bath at 37°C for 15 min and examined using flow cytometric analysis (ACEA NovoCyte 2040R, United States).

### Transfection of siRNA

Cells with 30–50% confluency were transfected with siRNA using Oligofectamine (Invitrogen). For each transfection, 200 nmols of siRNA was added per 100 mm plate (final concentration 40 nM). Two days after transfection cells were seeded into 6-well plates. Protein expression was verified by western blot.

### Western Blot Analysis

Cell lysates were collected at the indicated times in 0.1% NP-40 lysis buffer with complete protease inhibitor tablet (Roche, Basel, Switzerland), 1mM phenylmethanesulfonylfluoride (PMSF), and 2 mM sodium orthovanadate (New England BioLabs, Ipswich, MA, United States). Protein levels were quantified with a Pierce BCA kit (Thermo Fisher Scientific) according to the manufacturer’s instructions. Samples were run on 8–10% polyacrylamide gels and transferred onto nitrocellulose membranes (Bio-Rad, Hercules, CA, United States) blocked in 5% milk in TBS-T as the antibody manufacturer’s suggestions. Secondary antibodies were goat anti-rabbit-HRP or anti-mouse-HRP (Bio-Rad). Pierce ECL or Pierce Super signal Pico (Thermo Fisher Scientific) was used for the Detection of proteins.

### Transmission Electron Microscopy

The MDA-MB-231 cells were harvested and centrifuged at 1000 *g* for 5 min. Cells were immersed in 2.5% glutaraldehyde for 12 h at 4°C and then fixed in 1% osmium tetroxide for 1 h. After dyeing in 2% uranyl acetate for 1 h, samples were dehydrated in acetone and embedded in epoxy resin (Sigma, Epon 812). The 50–60 nanometer slices were made using Microtome (RMC boeckeler, PowerTome-XL), and collected on copper grids. Sections were stained with 2% uranyl acetate for 5 min and lead citrate for 1 min. Grids were subjected to a transmission electron microscope (Hitachi, H-7500) at 80 kV for observation of autolysosome.

### Confocal and Fluorescence Microscopy

Briefly, the MDA-MB-231 cell model expressing mRFP-LC3, mRFP-LC3, were cultured on cover slides in 10% FBS-supplemented DMEM. After synchronization, cells were cultured in complete medium for 12 h, followed by 0.1% FBS medium for another 12 h. The cells were fixed with 4% paraformaldehyde (Sigma Aldrich Corporation, 158127) in PBS (Gibco, 8117296) at room temperature (RT) for 15 min, followed by permeabilization with 0.25% Triton X-100 (Beyotime, ST795) at RT for 15 min and staining with LAMP2 (1:100; Cell Signaling Technology, D3U4 C) for 12 h at 4°C. The slides were washed three times with PBS and stained with corresponding secondary IgG for 60 min at RT. The slides were washed three times with PBS and once with ddH2O, and then fixed in glycerin. Cell images were captured using an inverted Nikon fluorescence microscope (A1 R). For the quantification of autophagic cells, puncta were determined in 20 random cells per slide.

### Statistical Analysis

All *in vitro* data were generated with at least three independent experiments. Each experiment in the cell death analysis was carried out by 3–6 replicates. The measurement data were represented as means ± SD (*n* ≥ 3). The normal distribution and variance homogeneity were measured then mean values were compared by Student’s *t*-test or repeated measure ANOVA with *P* < 0.05 being considered as statistical significance. All the statistical analyses were performed by using IBM SPSS Statistics (version 24.0; Armonk, NY, United States) and GraphPad Prism Version 8.0 software (GraphPad; San Diego, CA, United States).

## Results

### Radiation-Induced Autophagic Cell Death in MDA-MB-231 Breast Cancer Cells

**FIGURE 1:**
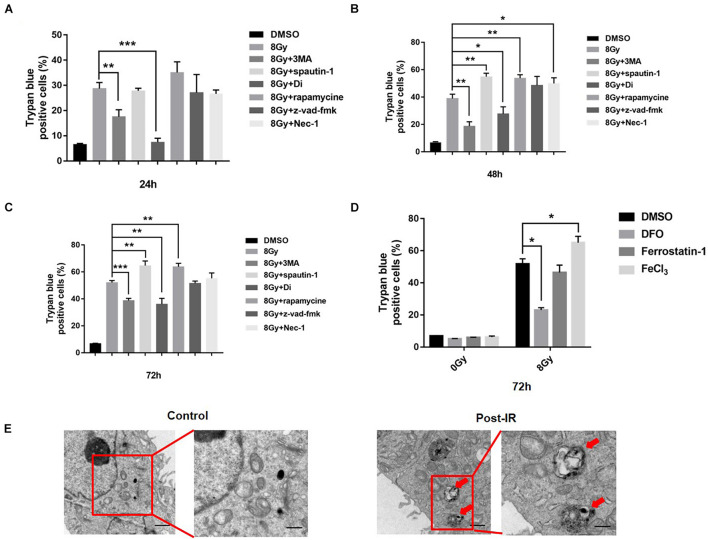
Radiation induced cell death in MDA-MB-231 breast cancer cells. MDA-MB-231 were pretreated with different inhibitors of cell death, i.e., 3MA (2 mM), spautin-1 (3 μM), rapamycin (5 nM), z-VAD-fmk (10 μM), Necrostain-1 (10 μM), Digitoxigenin (Di, 5 μM) and cell deaths were quantified at 24, 48, 72 h after radiation **(A–C)**. Cells were pretreated with FeCl3 (30 μM, pretreated for 3 h), DFO (0.1 mM), Ferrostain-1 (5 μm) for 1 h, cell death was quantified at 48 h after 8 Gy radiation **(D)**. Electron microscopy analysis of autophagosome in the MDA-MB-231 cells was made after radiation treatment. Red arrow pointed to autophagosomes **(E)**. These results were representative of three independent experiments and expressed as means ± SD. *P*-values were calculated using repeated measure ANOVA with *P* < 0.05 being considered as statistical significance, *P < 0.05, **P < 0.01, and ***P < 0.001.

To determine the main types of cell death at different time points following radiation treatment, we used regulators such as ferroptosis inhibitor Fer-1 autophagy inhibitor 3MA/Spautin-1/Digitoxigenin (Di), autophagy inducer Rapamycin, apoptosis inhibitor Z-VAD-FMK, and necroptosis inhibitor Necrostain-1 for pretreatment. Cell death was detected at 24, 48, and 72 h after 8 Gy radiation in MDA-MB-231(TNBC) (Figures 1A–C). The results showed that autophagy inhibitors 3MA and Di decreased radiation-induced cell death significantly, that is, from 31 to 18% or 10%, whereas rapamycin increased cell death from 31 to 40% following radiation treatment. Electron microscopy demonstrated the autophagosomes in MDA-MB-231 cells following radiation treatment, suggesting the participation of autophagy in radiation-induced cell death (Figure 1E). The iron chelator DFO decreased and FeCl_3_ increased radiation-induced cell death significantly from 48 to 20% and 48 to 65%, respectively (Figure 1D), suggesting the participation of iron in radiation-induced cell death. The apoptosis inhibitor Z-VAD-FMK failed to change cell death after radiation treatment. To confirm the Z-VAD-FMK activity, we used cisplatin to induce apoptosis, and Z-VAD-FMK was proven effective in decreasing cisplatin-induced cell death (Supplementary Figure 1). Radiation induced ferroptosis and apoptosis in breast cancer cells such as MCF-7 and zr-75 (estrogen receptor-positive), suggesting that radiation-induced cell death is cell type-dependent. We also treated the non-malignant epithelial mammary cell MCF-10A with radiation, and the cell survival rate failed to change at the same treatment (Supplementary Figure 2).

### Radiation Increased Iron Accumulation via Iron Regulatory Proteins

**FIGURE 2:**
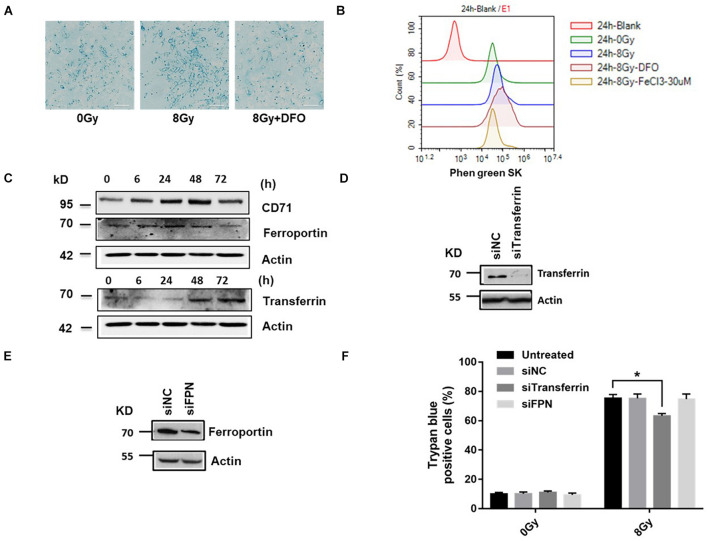
Radiation increased iron level and ROS generation in MDA-MB-231 breast cancer cells. Cells were pretreated with DFO, followed by 8 Gy radiation, Prussian blue staining for intracellular iron was performed **(A)**. Cells were pretreated with DFO or FeCl3 before 8 Gy radiation. Intracellular chelatable iron was detected using the fluorescent indicator Phen green SK with Flow cytometry **(B)**. The expressions of iron-related proteins Transferrin (Tf), Transferrin receptor (CD71), and Ferroportin (FPN) were performed after 8 Gy treatment **(C)**. Knockdown of Transferrin, FPN by siRNA as demonstrated by western blot **(D,E)**. Cells were transfected with control siRNA (siNC) and siRNA against Transferrin (si Tf), Ferroprotein (siFPN) for 48 h, the amount of cell death was determined at 48 h after 8 Gy radiation **(F)**. These results were representative of three independent experiments and expressed as means ± SD. *P*-values were calculated using repeated measure ANOVA with *P* < 0.05 being considered as statistical significance, *P < 0.05.

Intracellular iron chelator DFO decreased cell death, suggesting that iron affects radiation-induced cell death. We verified the role of iron in radiation-induced cell death. Data showed that radiation increased intracellular iron significantly at 24 h in MDA-MB-231 cells, DFO decreased iron levels (Figure 2A). Intracellular chelatable iron was also determined using the fluorescent indicator phen green SK, the fluorescence of which is quenched by free iron particles, compare to 8Gy radiation, DFO increased and FeCl_3_ decreased the fluorescence of phen green SK respectively, suggesting DFO decreased iron levels and FeCl_3_ increased iron level following 8 Gy treatment (Figure 2B). In addition, iron accumulation is actively regulated in cells through transferrin (Tf), transferrin receptor (CD71) and ferroportin (FPN) by importing iron into cells or exporting iron out of cells, respectively. To determine the effects of IR on iron regulatory proteins, Tf, CD71 and FPN was detected at 6, 24, 48, and 72 h after radiation. Data showed that after radiation, Tf and CD71 increased, while FPN decreased (Figure 2C), suggesting that the increase in intracellular iron might have been caused by iron import. Cell models were established by silencing of transferrin (siTf), ferroportin (siFPN), si CD71 and radiation-induced cell death decreased only by siTf (Figures 2D–F and Supplementary Figure 3). These data showed that alterations in iron transport proteins participated in iron-dependent cell death induced by radiation.

### Radiation Increased Iron Accumulation via Lysosomal Membrane Permeability

**FIGURE 3:**
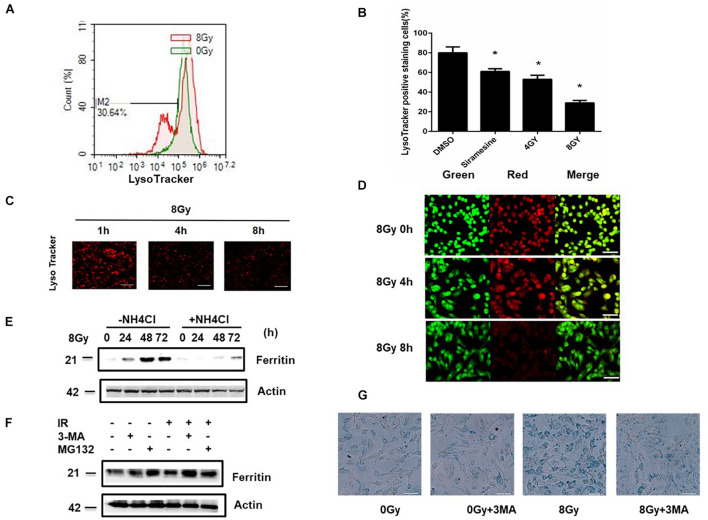
Radiation induced Lysosomal membrane permeabilization (LMP) in MDA-MB-231 breast cancer cells. MDA-MB-231 cells were treated with 4 Gy, 8 Gy radiation respectively, the amount of LMP was detected at 4, 8 h with LysoTracker and analyzed by flow cytometry **(A–C)** or with AO staining **(D)**. In the presence or absence of NH_4_Cl, the Ferritin (FTH) expression were detected after 8 Gy treatment at 24 h **(E)**. Cells were pretreated with 3MA (2 mM) or MG132 (1 μM) for 1 h before 8 Gy radiation, the Ferritin (FTH) expression were detected at 24 h **(F)**. Cells were pretreated with 3MA before 8 Gy radiation, the intracellular iron was evaluated with Prussian blue staining at 24 h by light microscopy **(G)**. These results were representative of three independent experiments and expressed as means ± SD. *P*-values were calculated using repeated measure ANOVA with *P* < 0.05 being considered as statistical significance, *P < 0.05.

It has been found that the continuous intralysosomal autophagocytotic degradation of ferruginous materials seems to produces the major pool of low-mass redox-active intracellular iron in the lysosomes. In the process, inside and outside formation of these organelles may cause lysosomal labialization with release to the cytosol of lytic enzymes and low-mass iron. LMP was detected by flow cytometry or by staining with Lyso Tracker or acridine orange. The amount of LMP increased by 39 and 62% following 4 and 8 Gy radiation compared to the sham radiation group. Siramesine is a lysosomal detergent as a positive control (Figures 3A–C). LMP causes the loss of the pH gradient over lysosomal membranes, and as a result, cells showed reduced staining with AO after 8 Gy treatment for 4 and 8 h (Figure 3D). This suggested that radiation-induced LMP is time- and dose- dependent. To determine whether LMP caused an increase in iron, the iron level was detected in the presence of NH_4_Cl (30 mM), which could elevate lysosomal pH and indirectly protect LMP. The iron level was found to decrease, suggesting that iron is part of the LMP. To determine whether iron-ROS feedback affects LMP, cells were pretreated with DFO and DFO failed to change the LMP, suggesting that LMP is partly due to lysosomal iron (Supplementary Figure 4). While pretreatment with the LMP inhibitor NH_4_Cl, ferritin expression significantly decreased, suggesting that ferritin induction is the downstream event of lysosomal rupture (Figure 3E). Ferritin degradation can occur through two different mechanisms, that is, lysosomal or proteasome. To determine ferritin degradation following radiation treatment, 3MA and MG132 were used for pretreatment. The results showed that 3MA prevented ferritin loss (Figure 3F) and 3MA significantly decreased radiation-induced iron levels (Figure 3G), confirming that iron was from autophagy degradation.

### Inhibition of Cathepsin L Failed to Block Radiation-Induced Cell Death

**FIGURE 4:**
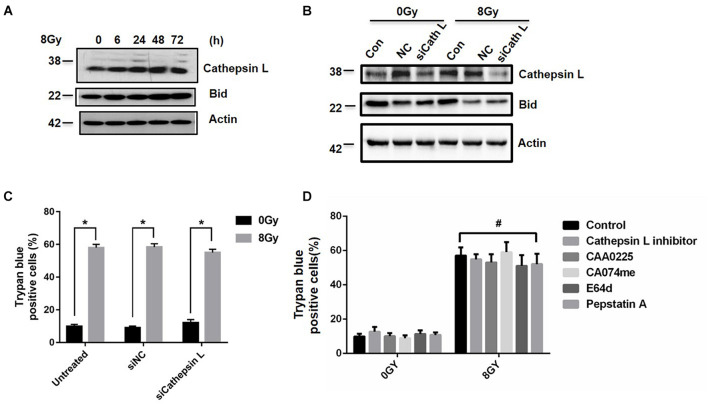
Inhibition of Cathepsin L fails to block radiation-induced cell death. Cathepsin L expression was analyzed by western blot in MDA-MB-231 cells at 6, 24, 48, 72 h after 8 Gy radiation **(A)**. After the knockdown of Cathepsin L by siRNAs, Cathepsin L and Bid expressions were determined after 8 Gy radiation **(B)**. Cells were untransfected or transfected with control siRNA (siNC) and siRNA against Cathepsin L (siCathepsin L) for 48 h before 8 Gy radiation, then cell death was determined **(C)**. Cells were pretreated with CAA0225 (a Cathepsin L inhibitor, 10 μg/ml) for 1 h before 8 Gy treatment, cathepsin L and Cleaved-Caspase3 expression were tested **(D)**. These results were representative of three independent experiments and expressed as means ± SD. *P*-values were calculated using repeated measure ANOVA with *P* < 0.05 being considered as statistical significance, *P < 0.05, #*P* > 0.05.

Since LMP could lead to the release of cathepsins and other hydrolases from the lysosomal lumen to the cytosol (Johansson et al., 2010), we examined whether cathepsins were involved in radiation-induced cell death. Cathepsin L expression increased at 6, 24, 48, and 72 h after 8 Gy treatment (Figure 4A), but cathepsin B and D failed to change (Supplementary Figure 5). After the knockdown of cathepsin L by siRNAs, cathepsin L and Bid expression decreased in the presence or absence of 8 Gy radiation (Figure 4B), while the amount of radiation-induced cell death did not change (Figure 4C). When cells were treated with radiation in the presence or absence of the cathepsins inhibitors as cathepsin L inhibitor (10 μM), CAA0225(10 μg/ml), CA074me (10 μM), E64d (10 μg/ml), Pepstatin A (4 μM), the amount of cell death did not change (Figure 4D). These results suggested that cathepsins failed to block radiation-induced cell death.

### Reactive Oxygen Species Generation Is Due in Part to Iron

**FIGURE 5:**
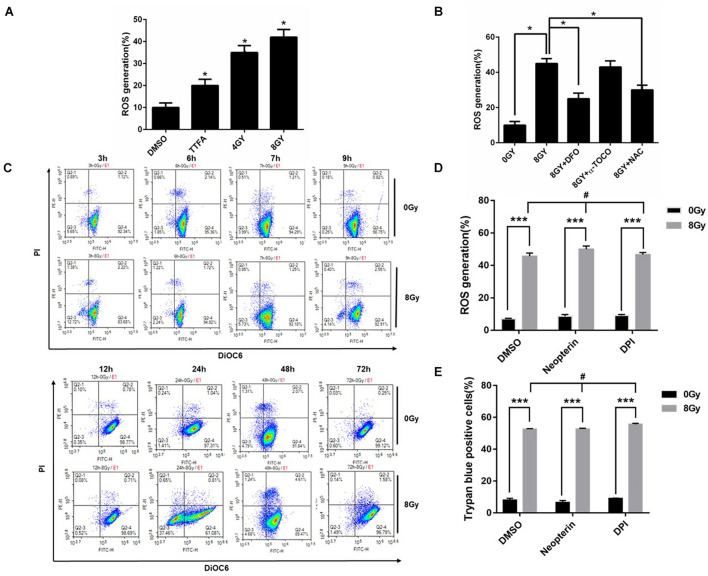
Radiation-induced ROS generation is due in part to iron in MDA-MB-231 breast cancer cells. Radiation-induced ROS was determined in MDA-MB-231 cells, TTFA (Hypochondria Complex II inhibitor) was used as a positive control **(A)**. The effects of DFO (0.1 mM), NAC (10 mM), and *a*-tocopherol (2 μM) on radiation-induced ROS in MDA-MB-231 cells was determined **(B)**. The mitochondrial transmembrane potential (ΔΨm) was detected with DIOC6(3)/PI and analyzed by flow cytometry **(C)**. Cells were pretreated with Neopterin (50 nM), DPI (5 μM) for 1 h before 8 Gy radiation, the amount of ROS generation and cell death was determined **(D,E)**. These results were representative of three independent experiments and expressed as means ± SD. *P*-values were calculated using repeated measure ANOVA with *P* < 0.05 being considered as statistical significance, *P < 0.05, ***P < 0.001, #*P* > 0.05.

The Fenton reaction involves the reaction of iron (Fe^2+^) with hydrogen peroxide (H_2_O_2_) to yield a hydroxyl radical (•OH) and hydroxide ion (OH^–^) (Dixon and Stockwell, 2014). The intracellular microenvironment of low pH and high iron content in lysosomes makes lysosomes an excellent source of ROS. Our results showed that radiation increased ROS generation at 48 h in MDA-MBHXO2-231 cells, while DFO, NAC decreased the ROS from 45 to 23%, 30% respectively (Figures 5A,B), suggesting the iron chelator was acting on the lysosomal production of ROS. ROS are also generated from the mitochondria and membrane NADPH oxidases. To demonstrate whether ROS generation is from mitochondria, intact mitochondria was quantified by flow cytometry using Mito tracker green. There was no change after treating with radiation over 24 h time course in MDA MB 231 cells. A DIOC6 dye was used to detect mitochondrial membrane potential over 72 h time course in MDA MB 231 cells, and there was only mitochondrial membrane potential drop at 24 h and quickly recovered at 72 h following 8 Gy radiation (Figure 5C and Supplementary Figure 6). To demonstrate whether ROS generation is from NADPH oxidases, ROS generation was measured after MDA-MB-231 cells were pretreated with NADPH oxidase inhibitor DPI and Neopterin. These inhibitors failed to change ROS levels and cell death following radiation treatment (Figures 5D,E). In summary, the results indicated that ROS generation was due to iron and it exerted a pivotal role in radiation-induced cell death.

### Reactive Oxygen Species Regulated Radiation-Induced Autophagic Cell Death

**FIGURE 6:**
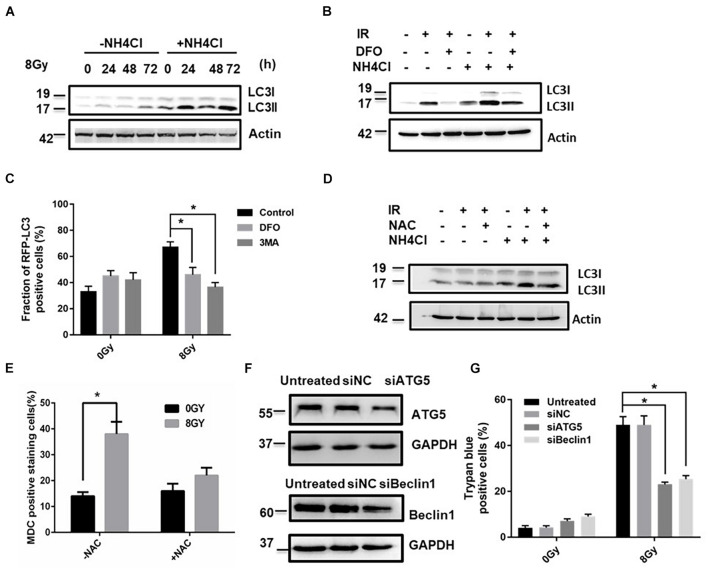
ROS regulated radiation-induced autophagic cell death in MDA-MB-231 breast cancer cells. The time-course analysis of autophagy marker MAPLC3-II by western blot in the absence and presence of NH4Cl after 8 Gy radiation **(A)**. The effects of DFO on the autophagy flux by western blot after 8 Gy radiation **(B)**. mRFP-LC3B puncta per cell were calculated after 8 Gy treatment **(C)**. The effects of NAC (10 mM) on the autophagy flux by western blot after 8 Gy radiation **(D)**. MDC staining was used to detect autophagy occurrence with flow cytometry analysis **(E)**. The autophagy genes Atg5 and Beclin-1 was knocked down by siRNAs for 48 h in MDA-MB-231 cells and the transfection efficiency was demonstrated by western blot **(F)**. The trypan blue assay shows the effects of silencing of Atg5 or Beclin-1 on radiation-induced cell death **(G)**. These results were representative of three independent experiments and expressed as means ± SD. *P*-values were calculated using repeated measure ANOVA with *P* < 0.05 being considered as statistical significance, *P < 0.05.

To determine whether ROS generation might induce autophagy, the autophagy-related protein MAPLC3-II was detected by western blotting in the absence and presence of the lysosomal inhibitor ammonium chloride (NH_4_Cl) (30 mM), and the expression of MAPLC3-II increased at 24, 48, and 72 h after 8Gy radiation (Figure 6A and Supplementary Figure 7). To determine whether iron affected autophagy flux, the autophagy-related protein MAPLC3-II was detected by western blotting in the absence and presence of DFO (100 μM) and NH_4_Cl (30 mM), DFO significantly decreased the expression of MAPLC3-II after 8 Gy radiation (Figures 6B,C). Similar results were observed in NAC treated cells. NAC significantly decreased radiation-induced autophagy from 38 to 21% (Figures 6D,E), suggesting that iron-induced ROS generation was involved in the process of autophagy. Knockdown of autophagy relative genes Atg5 and Beclin1 by siRNAs reduced radiation-induced cell death at 48 h from 48 to 20% and 27% (Figures 6F,G), respectively. This suggested that radiation-induced autophagy functioned as a pro-death pathway in an Iron dependent manner.

### Superoxide Dismutase (SOD2) Decreased Autophagy and Cell Death After Radiation Treatment

**FIGURE 7:**
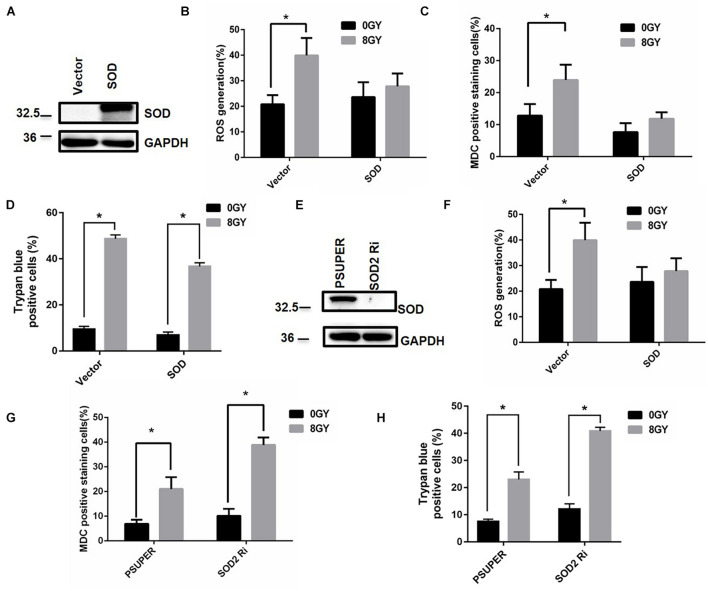
Superoxide dismutase (SOD2) decreased autophagy and cell death after radiation treatment. Cells were transfected with control vector (Vector) and SOD2 overexpression plasmid (SOD) for 24 h, the SOD2 expression was tested by Western blot **(A)**. Intracellular ROS **(B)** autophagic rate **(C)** and the amount of cell death **(D)** were determined at 48 h after radiation treatment. Cells were transfected with control vector (pSUPER) and siRNA against SOD2 (SOD2 Ri) for 48 h, the SOD2 expression was tested by Western blot **(E)**. Intracellular ROS **(F)**, autophagic rate **(G)**, and the amount of cell death **(H)** were determined at 48 h after radiation treatment. These results were representative of three independent experiments and expressed as means ± SD. *P*-values were calculated using repeated measure ANOVA with *P* < 0.05 being considered as statistical significance, *P < 0.05.

The mitochondrial superoxide dismutase 2 (SOD2) enzyme is critical in the metabolism of superoxide (Yang et al., 2006). To determine whether SOD2 affects ROS generation, autophagy, and cell death after radiation treatment, the control vector and SOD2 cDNA plasmid were transfected into MDA-MB-231 cells (Figure 7A). The results showed that radiation-induced ROS generation decreased upon overexpression of SOD2. Similarly, radiation-induced autophagy decreased by overexpression of SOD2 from approximately 25 to 12% (Figures 7B–D), and radiation-induced cell death decreased from 50 to 38%. The above results indicated that overexpression of SOD2 could prevent ROS generation, autophagy, and cell death. Conversely, the expression of SOD2 was suppressed by transfection of MDA-MB-231 cells (wild type) with siRNA against SOD2 (Figure 7E), radiation induced an increase in ROS generation, autophagy, and cell death from 32 to 50%, 20 to 40%, and 20 to 38% by silencing of SOD2, respectively (Figures 7F–H). The above results indicated that suppression of SOD2 elevated ROS generation, autophagy, and cell death.

### Blockage of Autophagy Failed to Block Radiation-Induced Reactive Oxygen Species Generation in Breast Cancer Cells

Whether or not the process of autophagy could regulate ROS generation with positive feedback is our next question. The knockdown of Atg5 or Beclin-1, the pretreatment with 3MA failed to block radiation-induced ROS generation by using DHE or MitoSOX staining following 8Gy in MDA-MB-231 cells, indicated that ROS generation is upstream of autophagy (Supplementary Figure 8).

### A Schematic Model Demonstrating the Roles of Iron in Radiation-Induced Cell Death

**FIGURE 8:**
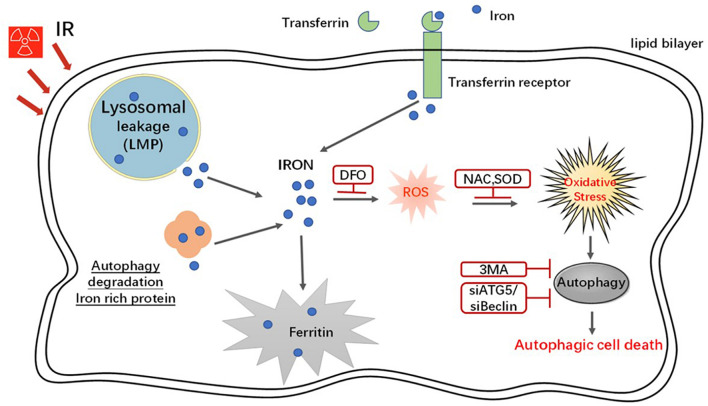
A schematic model demonstrating the roles of iron in radiation-induced cell death. In an *in vitro* system, radiation increased the iron levels via the increase of the expression of iron regulatory proteins, breakage LMP and autophagy degradation iron storage protein. Iron increased intracellular ROS through Fenton reaction and furthermore caused autophagy and autophagic cell death.

In an *in vitro* system, radiation increased iron levels via an increase in the expression of iron regulatory proteins, breakage of LMP, and autophagy degradation of iron storage protein. Iron increased ROS generation through the Fenton reaction and caused autophagic cell death (Figure 8).

## Discussion

Radiation can induce cell death depending on cell type and radiation dose, and different types of cell death might exist at the same time, but they contribute differently (Golden et al., 2012). To determine the main type of cell death following radiation treatment in MDA-MB-231 cells, several inducers or inhibitors of cell death were used in this study. Our results show that 3MA significantly decreases radiation-induced cell death, while Z-VAD-FMK and Necrostatin-1 fail to protect cells from death, suggesting that autophagy might be predominant and contribute to cell death under these circumstances. It has been demonstrated that the ROS generated by the aqueous reaction of Fe^2+^ and H_2_O_2_ (i.e., the Fenton reaction) are involved in radiation damage. Several antioxidants and regulators were used including an iron chelator DFO, which significantly decreased radiation-induced cell death, suggesting that iron might be involved. The data shows that DFO, NAC, or overexpression of SOD2 led to the downregulation of radiation-induced ROS and autophagy. Treatment with 3MA or siRNAs against beclin-1 or Atg 5 failed to block ROS production, suggesting ROS generation is the upstream of autophagy.

In addition, there is a clear division of labor between intracellular transferrin and FPN that transferrin transports iron into cells while FPN exports iron out of Ward and Kaplan (2012). We investigated whether iron regulatory proteins such as transferrin and FPN would contribute to cell death. So, transferrin, transferrin receptor, and FPN protein were detected at 6, 24, 48, and 72 h after radiation. The expression of FPN decreased after radiation. In contrast, the expression of transferrin significantly increased by radiation in MDA-MB-231 cells, and silencing transferrin rescued the cell death. These results indicated that radiation treatment altered iron transport and led to intracellular iron accumulation, ultimately increasing cell death.

Recently, LMP has been verified to initiate a cell death pathway in certain conditions. Lysosomes are important organelles for the degradation of proteins and the occurrence of autophagy. The degradation of iron-containing proteins could lead to the accumulation of iron in lysosomes (Ghosh et al., 2011; Ma et al., 2016, 2017). In general, lysosomal leakage leads to the release of small molecules, hydrolysis of cytosolic proteins and phospholipids, and the subsequent cascade reaction is mainly dependent on the hydrolysis substrates (Mrschtik and Ryan, 2015). What will be the results of LMP and how does the LMP contribute to cell death following radiation treatment? Our data showed the appearance of LMP after radiation and resulted in early upregulation of lysosomal cathepsins and intracellular iron but cathepsins failed to change the radiation-induced cell death, and the enhanced iron production was a late event in comparison with LMP. The results suggest that the radiation initially caused LMP, followed by the release of lysosomal iron, which generated ROS by the Fenton reaction. ROS further amplified LMP as feedback.

Autophagy is also well-known to play a vital role in maintaining physiological iron balance through the degradation of the iron storage protein ferritin (Krishan et al., 2015). Ferritin is a universal intracellular protein that stores iron and releases it in a manageable fashion (Munro and Linder, 1978). In humans, it acts as a buffer pool against iron deficiency and iron overload. Studies using the DFO have demonstrated that ferritin was degraded in lysosomes and the iron release was dependent on ferritin degradation. Excessive consumption of the cytosolic iron by the administration of the iron exporter FPN indicated that iron was released from ferritin shells in the cytosol (Domenico et al., 2006; de Domenico et al., 2009). Degradation of ferritin has been reported in cells suffering from amino acid starvation-induced autophagy (Kaur and Debnath, 2015). Some research groups reported that an increase in cellular iron might induce transcriptional upregulation of endogenous ferritin, and ferritin induction was the downstream event of lysosomal rupture (Torti and Torti, 2002). Since intracellular iron homeostasis is regulated by ferritin, when the cells are in an iron overload state, ferritin protein synthesis increases to store more iron and protect cells from oxidative damage. In this study, when cells were pretreated with the lysosomal weak base NH_4_Cl, the radiation-induced ferritin upregulation was blocked in MDA-MB-231 cells. The findings of the present study demonstrate for the first time that the induction of ferritin can be prevented by blockage of lysosomal rupture after radiation.

Theoretically, iron ions increase the production of cellular ROS via the Fenton reaction (Lin et al., 2010). Excessive production of ROS perturbs the redox balance and leads to oxidative damage (Ghosh et al., 2011). To overcome oxidative stress, cells possess different antioxidant enzyme systems, including superoxide dismutase, catalase, and glutathione systems (Aruoma et al., 1989). Our data showed that both antioxidant NAC and iron chelator DFO significantly reduced ROS levels, but other antioxidants (A-tocopherol, lycopene, fructose, and GSH) failed to change ROS levels. Since ROS can also be generated from the membrane NADPH oxidases (Li et al., 2017), the NADPH oxidase inhibitor DPI and neopterin failed to change ROS generation after radiation. The above-mentioned data indicates that ROS was mainly from iron.

The tumor microenvironment (TME) consists of numerous immune cells, mesenchymal cells, extracellular matrix, and active mediators (e.g., cytokines, chemokines, growth factors, and humoral factors) other than tumor cells. The main characteristics of TME, including hypoxia, acidic pH, and high ROS, also stimulate tumor autophagy. It is now widely accepted that the oxygen consumption of solid tumors is increased due to the tumor volume and elevation of the respiratory activity of different cell populations within a tumor. The increase in the oxygen consumption leads to the establishment of hypoxic TME. Ionizing radiation (IR) can induce damage in biological tissues and thus efficiently kill tumor cells. The accumulation of ROS and iron ions leads to the establishment of high ROS and high iron level TME. The tumor cells are probably inhibited and killed through iron-dependent autophagy in the aberrant TME.

To summarize, we found that radiation-induced autophagic death was iron-dependent in breast cancer MDA-MB-231 and BT549 cells (Supplementary Figure 9). These results provided new insights into the cell death process of cancers and might conduce to the development and application of novel therapeutic strategies for patients with apoptosis-resistant breast cancer.

## Data Availability Statement

The raw data supporting the conclusions of this article will be made available by the authors, without undue reservation.

## Author Contributions

SM and XDL conceived and designed the study. XF, LL, HF, HJ, XML, RL, ZL, ML, ZT, BH, and BL collected and analyzed the data. YB interpreted the data. SM wrote the manuscript. XDL reviewed and edited the manuscript. All the authors read and approved the final manuscript.

## Conflict of Interest

The authors declare that the research was conducted in the absence of any commercial or financial relationships that could be construed as a potential conflict of interest.

## Publisher’s Note

All claims expressed in this article are solely those of the authors and do not necessarily represent those of their affiliated organizations, or those of the publisher, the editors and the reviewers. Any product that may be evaluated in this article, or claim that may be made by its manufacturer, is not guaranteed or endorsed by the publisher.

## Abbreviations

LMP: lysosomal membrane permeabilization
ROS: reactive oxygen species
DFO: deferoxamine
NCCD: the Nomenclature Committee on Cell Death
FTH: ferritin
Tf: transferrin
FPN: ferroportin
NAC: *N*-acetylcysteine
H_2_O_2_: hydrogen peroxide
OH: hydroxyl free radicals
IR: ionizing radiation
ATCC: American type culture collection
AO: acridine orange
DPBS: Dulbecco’s phosphate-buffered saline
DHE: dihydroethidium
2-HE: 2-hydroxyethidium
MMP: mitochondrial membrane permeabilization
Δψ m: mitochondrial membrane potential
PMSF: phenylmethanesulfonylfluoride
Di: digitoxigenin
Fer-1: ferrostatin-1
RT: room temperature
Tf: transferrin
OH^–^: hydroxideion
SOD2: superoxide dismutase 2
HOCl: hypochloric acid
MDC: monodansylcadaverine.
