# Supplementary material for: Iron-Dependent Autophagic Cell Death Induced by Radiation in MDA-MB-231 Breast Cancer Cells
Source: Front Cell Dev Biol. 2021 Oct 14;9:723801. doi: 10.3389/fcell.2021.723801 (PMC8551752; doi:10.3389/fcell.2021.723801)
Supplement: Supplementary file 1 [file Presentation_1.PPTX]

## Slide 1
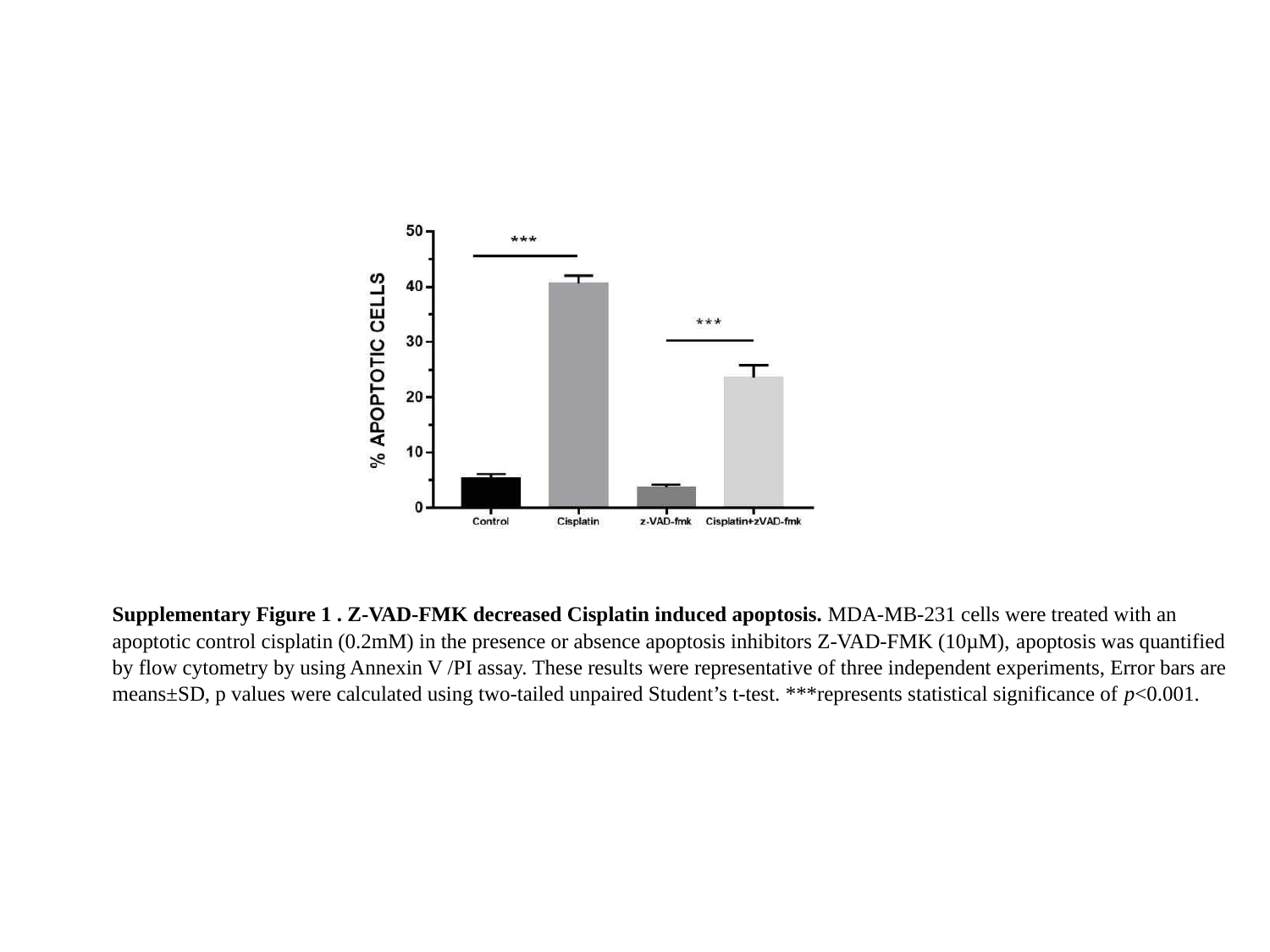

Supplementary Figure 1 . Z-VAD-FMK decreased Cisplatin induced apoptosis. MDA-MB-231 cells were treated with an apoptotic control cisplatin (0.2mM) in the presence or absence apoptosis inhibitors Z-VAD-FMK (10µM), apoptosis was quantified by flow cytometry by using Annexin V /PI assay. These results were representative of three independent experiments, Error bars are means±SD, p values were calculated using two-tailed unpaired Student’s t-test. ***represents statistical significance of p<0.001.

## Slide 2
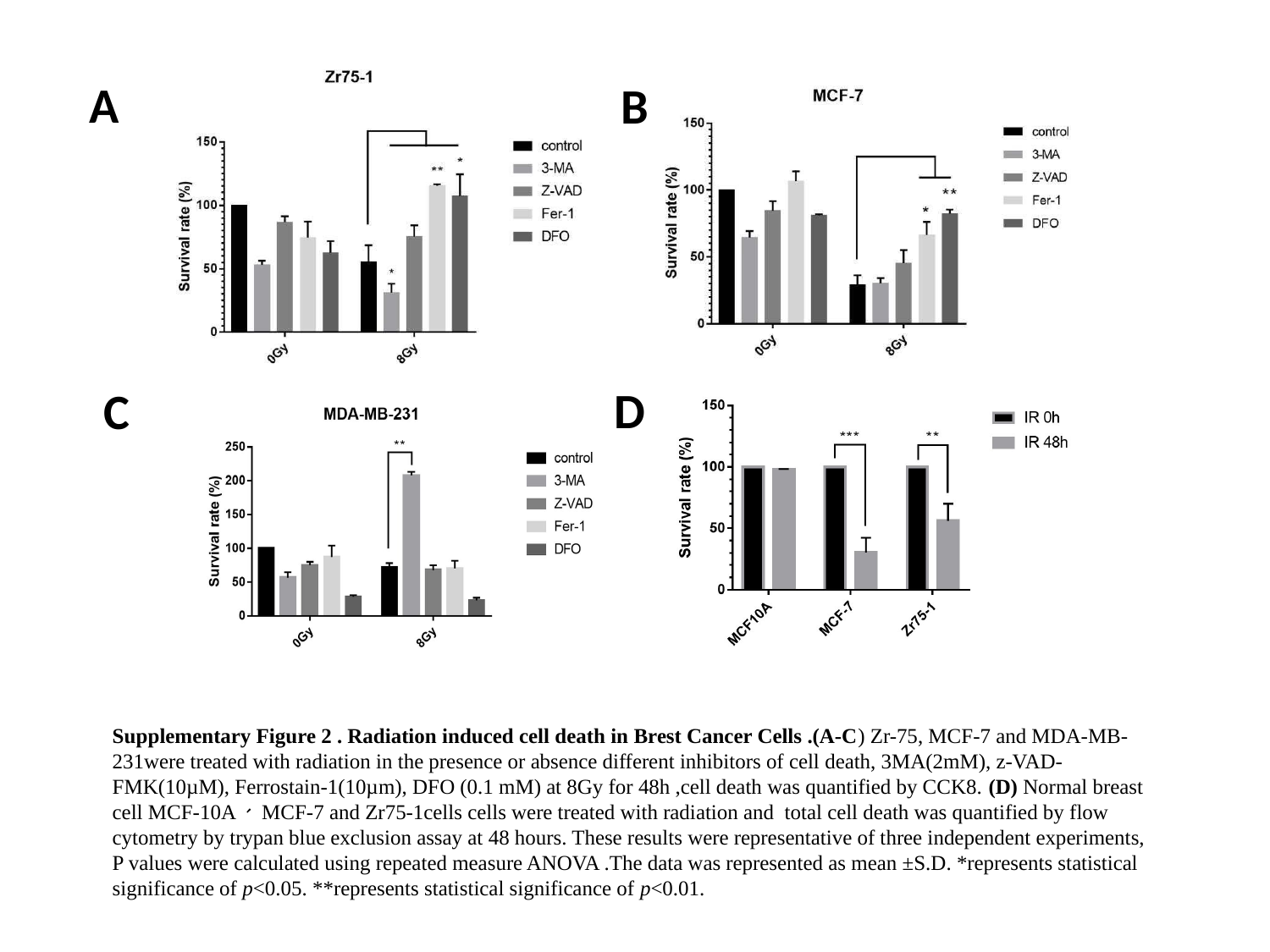

A
B
D
C
Supplementary Figure 2 . Radiation induced cell death in Brest Cancer Cells .(A-C) Zr-75, MCF-7 and MDA-MB-231were treated with radiation in the presence or absence different inhibitors of cell death, 3MA(2mM), z-VAD-FMK(10µM), Ferrostain-1(10µm), DFO (0.1 mM) at 8Gy for 48h ,cell death was quantified by CCK8. (D) Normal breast cell MCF-10A、 MCF-7 and Zr75-1cells cells were treated with radiation and total cell death was quantified by flow cytometry by trypan blue exclusion assay at 48 hours. These results were representative of three independent experiments, P values were calculated using repeated measure ANOVA .The data was represented as mean ±S.D. *represents statistical significance of p<0.05. **represents statistical significance of p<0.01.

## Slide 3
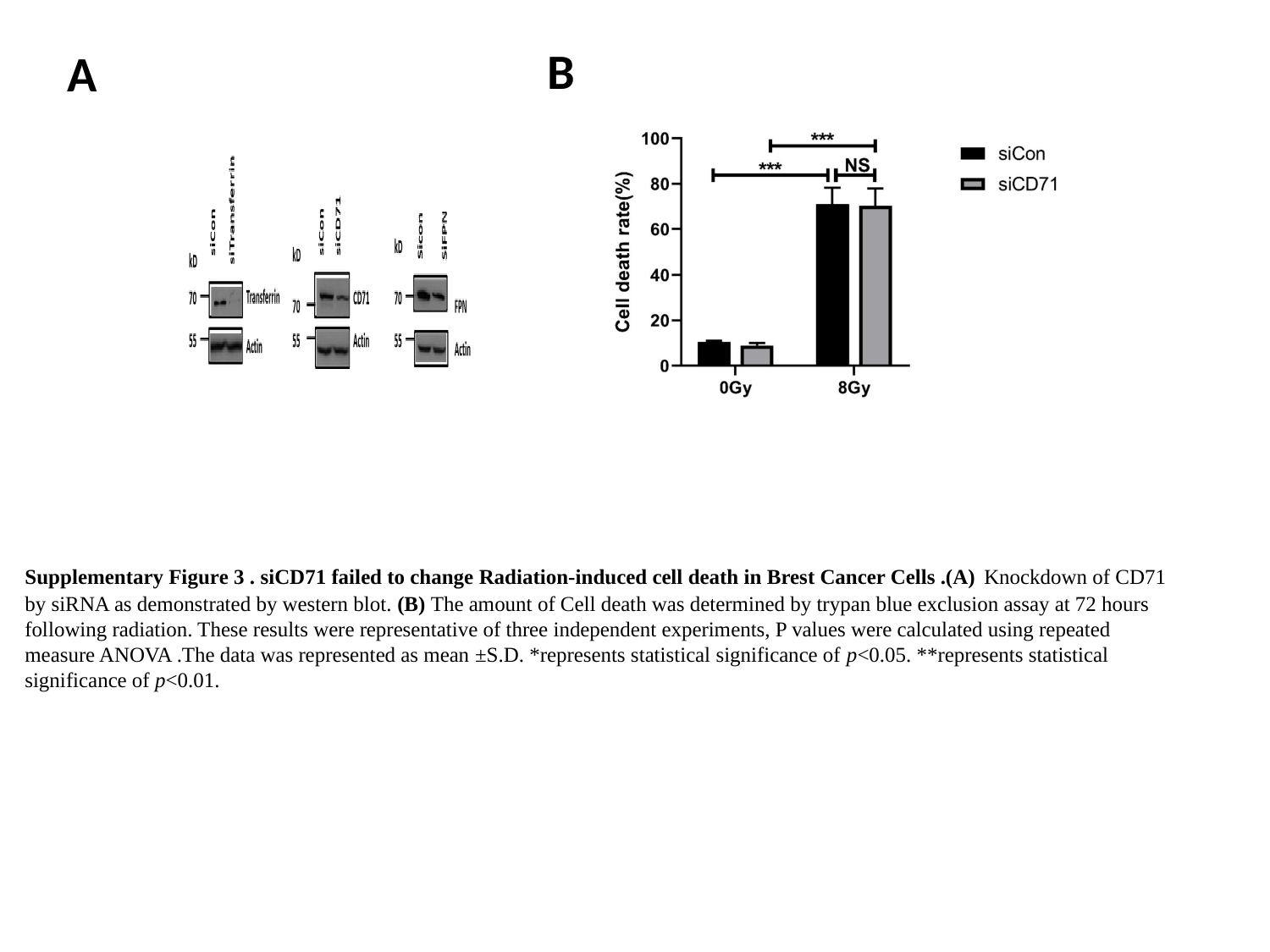

B
A
Supplementary Figure 3 . siCD71 failed to change Radiation-induced cell death in Brest Cancer Cells .(A) Knockdown of CD71 by siRNA as demonstrated by western blot. (B) The amount of Cell death was determined by trypan blue exclusion assay at 72 hours following radiation. These results were representative of three independent experiments, P values were calculated using repeated measure ANOVA .The data was represented as mean ±S.D. *represents statistical significance of p<0.05. **represents statistical significance of p<0.01.

## Slide 4
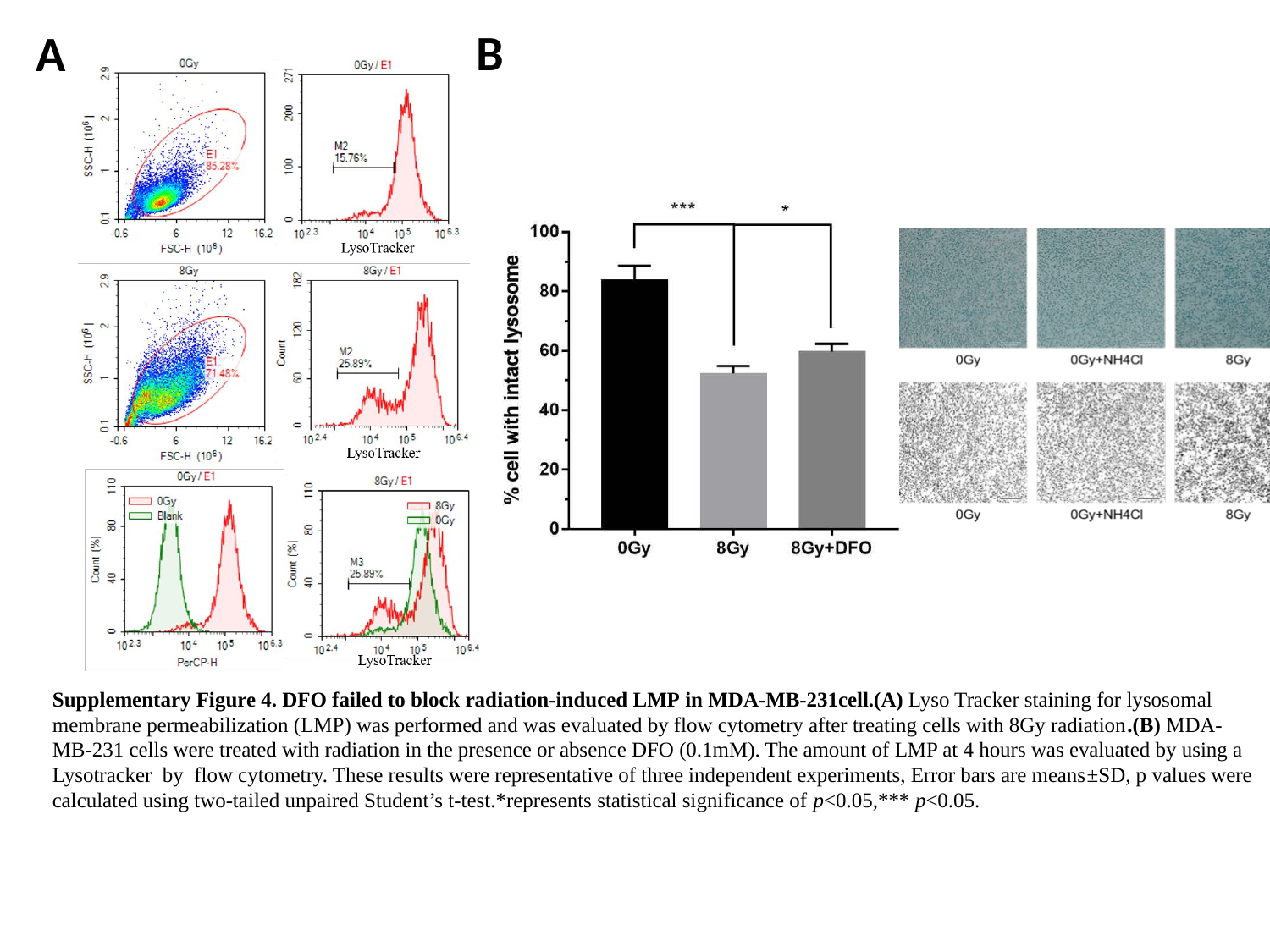

B
A
Supplementary Figure 4. DFO failed to block radiation-induced LMP in MDA-MB-231cell.(A) Lyso Tracker staining for lysosomal membrane permeabilization (LMP) was performed and was evaluated by flow cytometry after treating cells with 8Gy radiation.(B) MDA-MB-231 cells were treated with radiation in the presence or absence DFO (0.1mM). The amount of LMP at 4 hours was evaluated by using a Lysotracker by flow cytometry. These results were representative of three independent experiments, Error bars are means±SD, p values were calculated using two-tailed unpaired Student’s t-test.*represents statistical significance of p<0.05,*** p<0.05.

## Slide 5
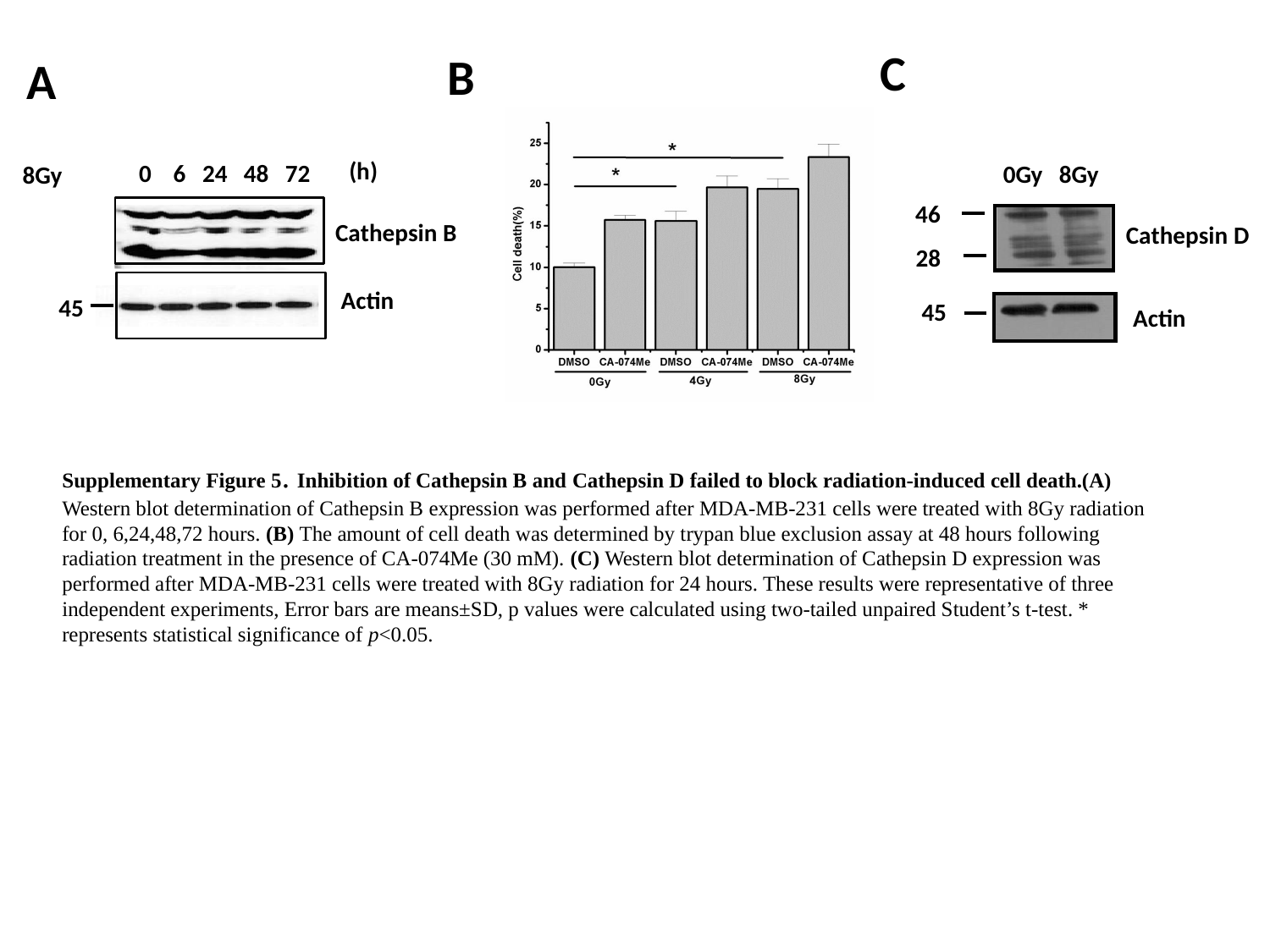

C
B
A
(h)
0 6 24 48 72
8Gy
Cathepsin B
Actin
0Gy 8Gy
46
Cathepsin D
28
Actin
45
45
Supplementary Figure 5. Inhibition of Cathepsin B and Cathepsin D failed to block radiation-induced cell death.(A) Western blot determination of Cathepsin B expression was performed after MDA-MB-231 cells were treated with 8Gy radiation for 0, 6,24,48,72 hours. (B) The amount of cell death was determined by trypan blue exclusion assay at 48 hours following radiation treatment in the presence of CA-074Me (30 mM). (C) Western blot determination of Cathepsin D expression was performed after MDA-MB-231 cells were treated with 8Gy radiation for 24 hours. These results were representative of three independent experiments, Error bars are means±SD, p values were calculated using two-tailed unpaired Student’s t-test. * represents statistical significance of p<0.05.

## Slide 6
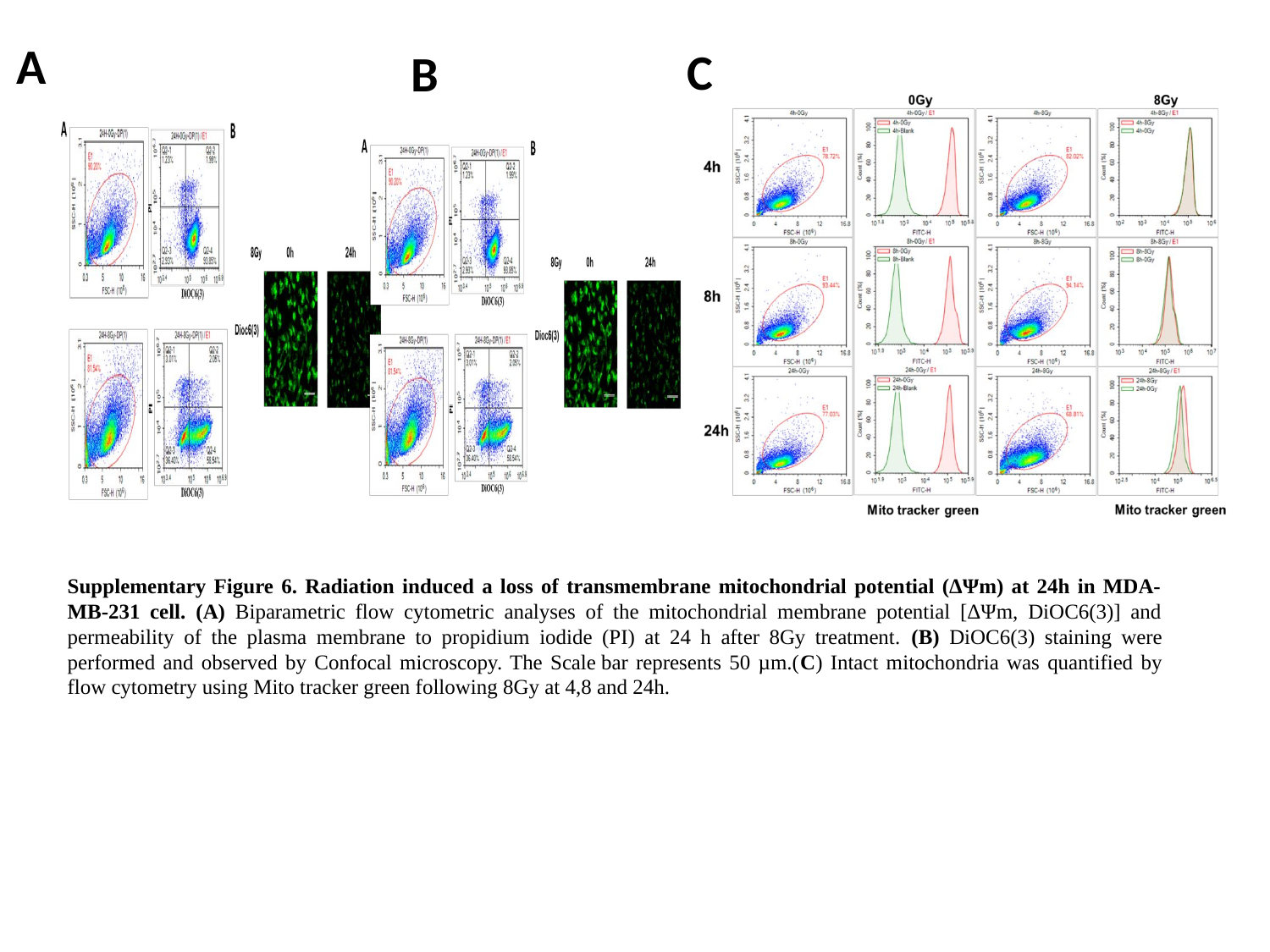

A
C
B
Supplementary Figure 6. Radiation induced a loss of transmembrane mitochondrial potential (ΔΨm) at 24h in MDA-MB-231 cell. (A) Biparametric flow cytometric analyses of the mitochondrial membrane potential [ΔΨm, DiOC6(3)] and permeability of the plasma membrane to propidium iodide (PI) at 24 h after 8Gy treatment. (B) DiOC6(3) staining were performed and observed by Confocal microscopy. The Scale bar represents 50 µm.(C) Intact mitochondria was quantified by flow cytometry using Mito tracker green following 8Gy at 4,8 and 24h.

## Slide 7
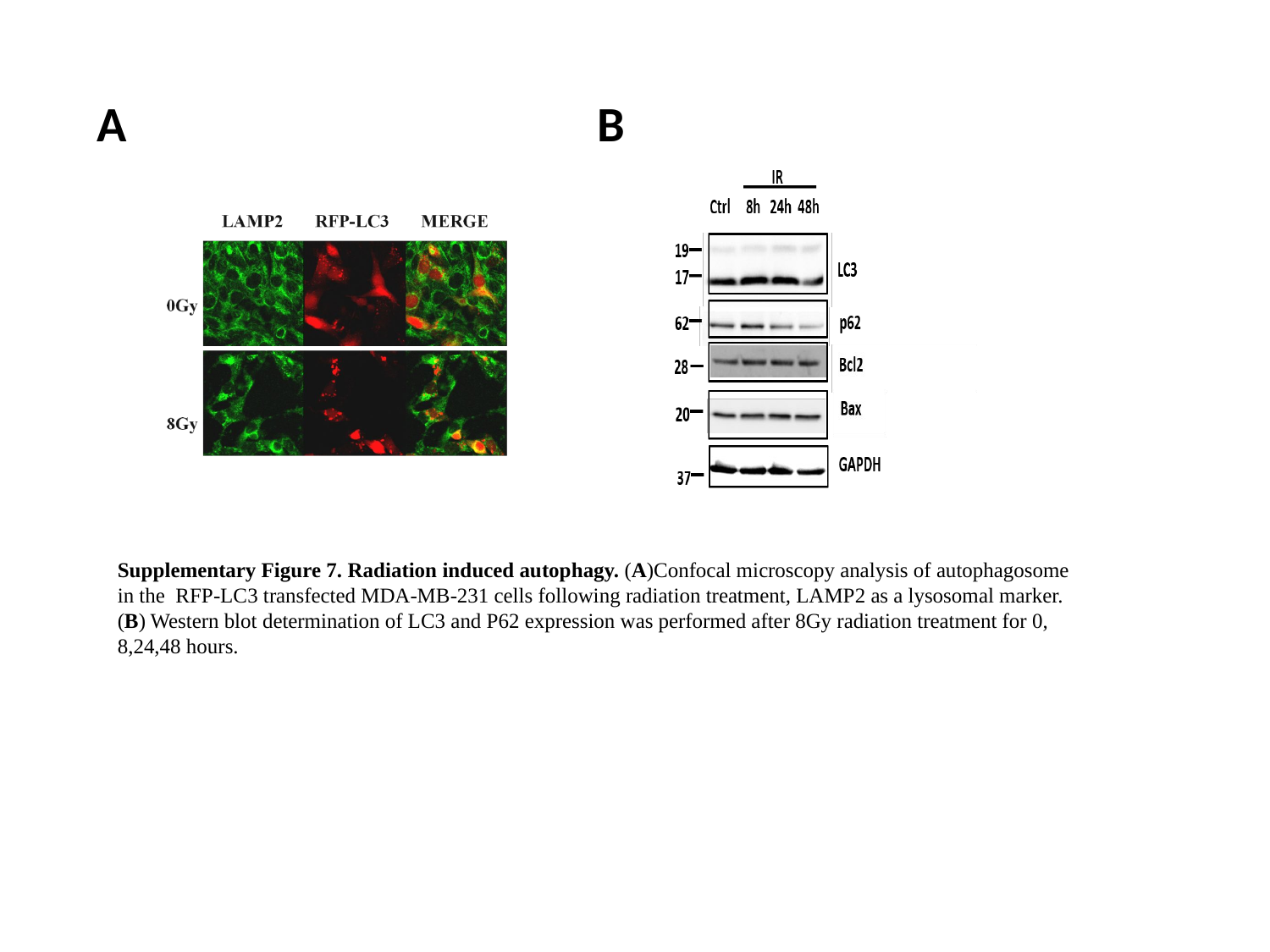

A
B
Supplementary Figure 7. Radiation induced autophagy. (A)Confocal microscopy analysis of autophagosome in the RFP-LC3 transfected MDA-MB-231 cells following radiation treatment, LAMP2 as a lysosomal marker. (B) Western blot determination of LC3 and P62 expression was performed after 8Gy radiation treatment for 0, 8,24,48 hours.

## Slide 8
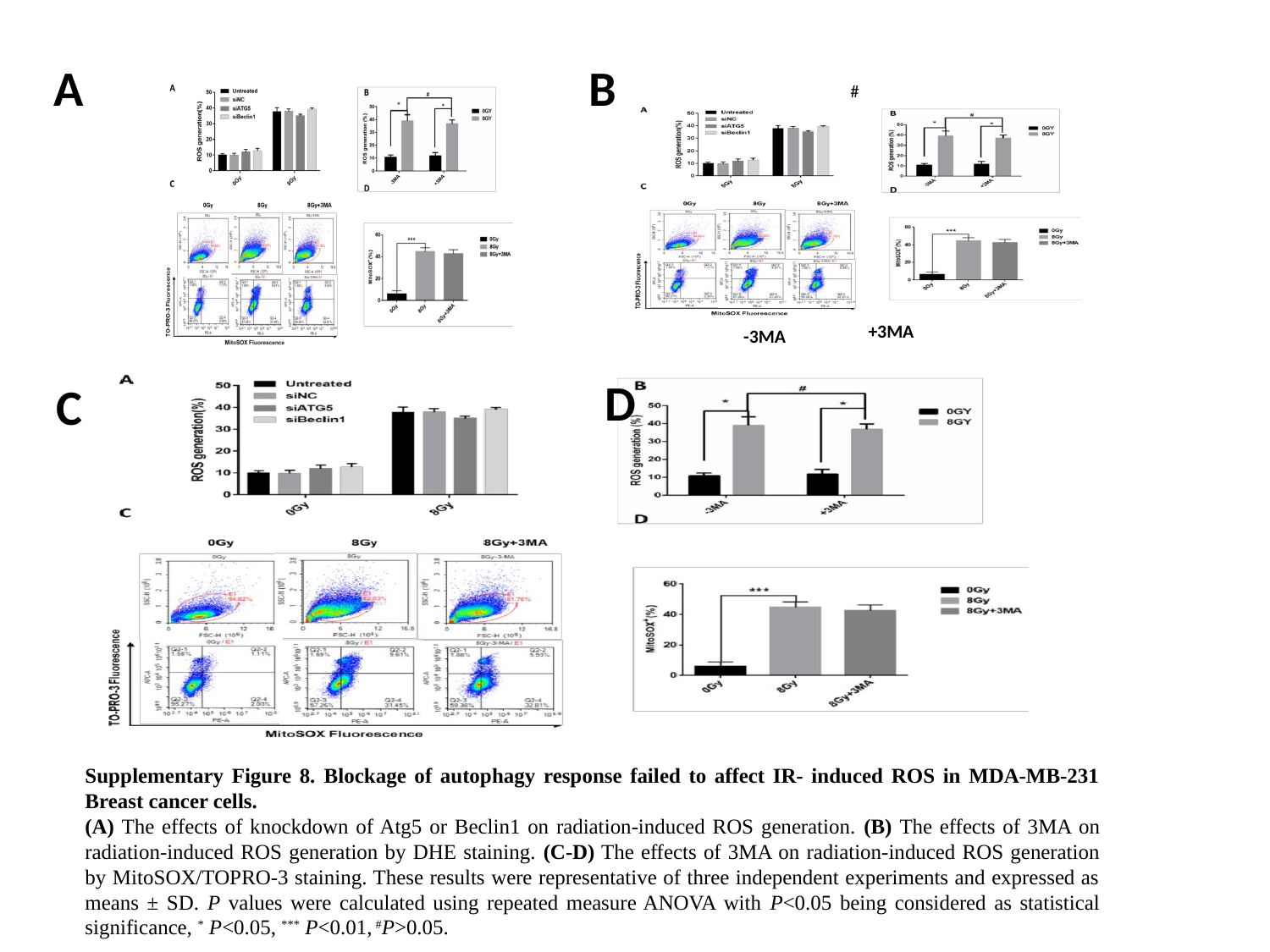

A
B
#
+3MA
-3MA
D
C
Supplementary Figure 8. Blockage of autophagy response failed to affect IR- induced ROS in MDA-MB-231 Breast cancer cells.
(A) The effects of knockdown of Atg5 or Beclin1 on radiation-induced ROS generation. (B) The effects of 3MA on radiation-induced ROS generation by DHE staining. (C-D) The effects of 3MA on radiation-induced ROS generation by MitoSOX/TOPRO-3 staining. These results were representative of three independent experiments and expressed as means ± SD. P values were calculated using repeated measure ANOVA with P<0.05 being considered as statistical significance, * P<0.05, *** P<0.01, #P>0.05.

## Slide 9
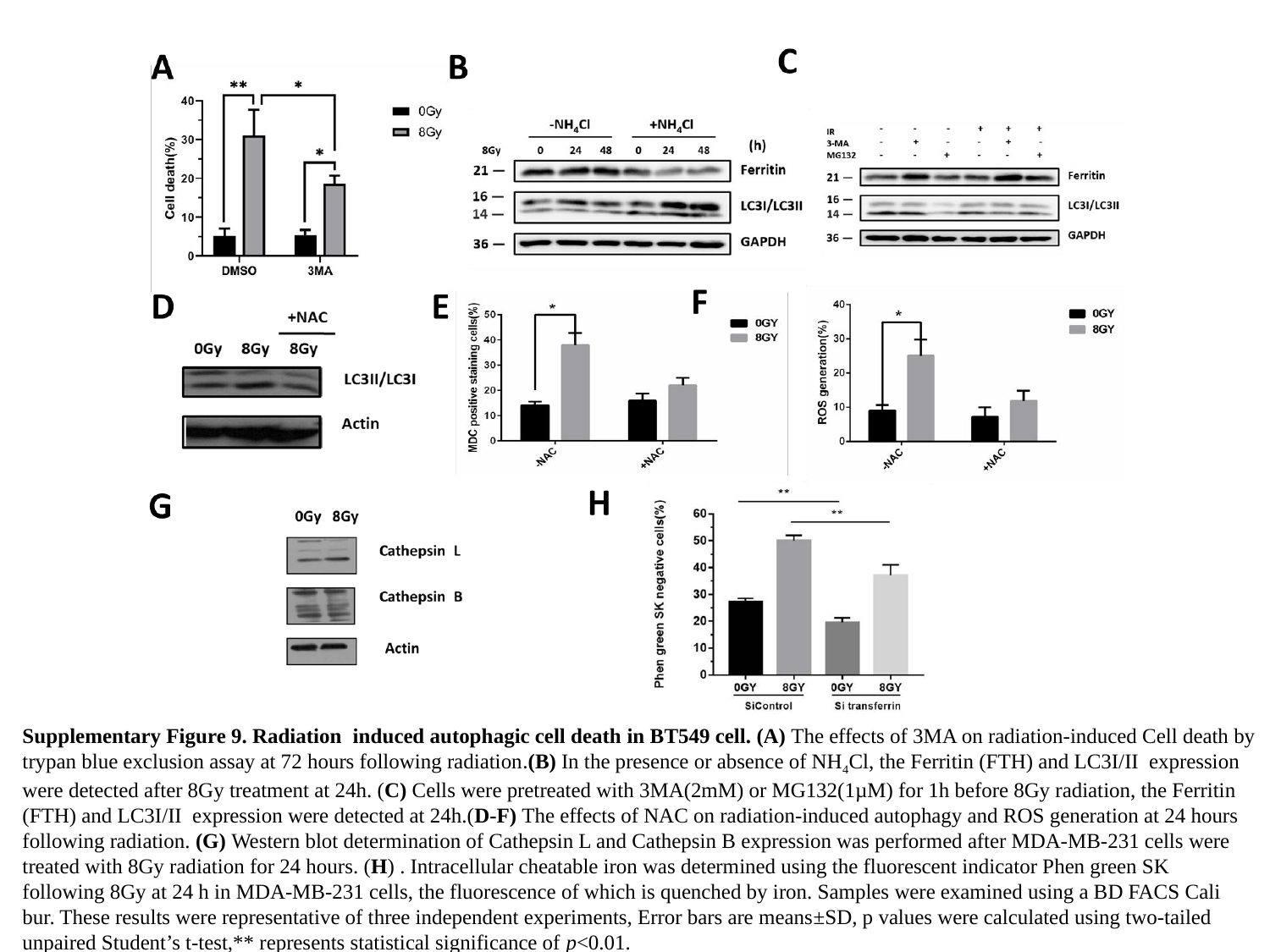

Supplementary Figure 9. Radiation induced autophagic cell death in BT549 cell. (A) The effects of 3MA on radiation-induced Cell death by trypan blue exclusion assay at 72 hours following radiation.(B) In the presence or absence of NH4Cl, the Ferritin (FTH) and LC3I/II expression were detected after 8Gy treatment at 24h. (C) Cells were pretreated with 3MA(2mM) or MG132(1µM) for 1h before 8Gy radiation, the Ferritin (FTH) and LC3I/II expression were detected at 24h.(D-F) The effects of NAC on radiation-induced autophagy and ROS generation at 24 hours following radiation. (G) Western blot determination of Cathepsin L and Cathepsin B expression was performed after MDA-MB-231 cells were treated with 8Gy radiation for 24 hours. (H) . Intracellular cheatable iron was determined using the fluorescent indicator Phen green SK following 8Gy at 24 h in MDA-MB-231 cells, the fluorescence of which is quenched by iron. Samples were examined using a BD FACS Cali bur. These results were representative of three independent experiments, Error bars are means±SD, p values were calculated using two-tailed unpaired Student’s t-test,** represents statistical significance of p<0.01.
